# Comparison of differentially expressed genes in longissimus dorsi muscle of Diannan small ears, Wujin and landrace pigs using RNA-seq

**DOI:** 10.3389/fvets.2023.1296208

**Published:** 2024-01-05

**Authors:** Qiuyan Li, Meilin Hao, Junhong Zhu, Lanlan Yi, Wenjie Cheng, Yuxiao Xie, Sumei Zhao

**Affiliations:** ^1^Faculty of Animal Science and Technology, Yunnan Agricultural University, Kunming, China; ^2^College of Biology and Agriculture, Zunyi Normal University, Zunyi, China

**Keywords:** transcriptome sequencing (RNA-seq), longissimus dorsi muscle, differentially expressed genes, Diannan small ears pig, Wujin pig, landrace pig

## Abstract

**Introduction:**

Pig growth is an important economic trait that involves the co-regulation of multiple genes and related signaling pathways. High-throughput sequencing has become a powerful technology for establishing the transcriptome profiles and can be used to screen genome-wide differentially expressed genes (DEGs). In order to elucidate the molecular mechanism underlying muscle growth, this study adopted RNA sequencing (RNA-seq) to identify and compare DEGs at the genetic level in the longissimus dorsi muscle (LDM) between two indigenous Chinese pig breeds (Diannan small ears [DSE] pig and Wujin pig [WJ]) and one introduced pig breed (Landrace pig [LP]).

**Methods:**

Animals under study were from two Chinese indigenous pig breeds (DSE pig, *n* = 3; WJ pig, *n* = 3) and one introduced pig breed (LP, *n* = 3) were used for RNA sequencing (RNA-seq) to identify and compare the expression levels of DEGs in the LDM. Then, functional annotation, Gene Ontology (GO) enrichment analysis, Kyoto Encyclopedia of Genes and Genomes (KEGG) pathway enrichment analysis, and Protein–Protein Interaction (PPI) network analysis were performed on these DEGs. Then, functional annotation, Gene Ontology (GO) enrichment analysis, Kyoto Encyclopedia of Genes and Genomes (KEGG) pathway enrichment analysis, and Protein-Protein Interaction (PPI) network analysis were performed on these DEGs.

**Results:**

The results revealed that for the DSE, WJ, and LP libraries, more than 66, 65, and 71 million clean reads were generated by transcriptome sequencing, respectively. A total of 11,213 genes were identified in the LDM tissue of these pig breeds, of which 7,127 were co-expressed in the muscle tissue of the three samples. In total, 441 and 339 DEGs were identified between DSE vs. WJ and LP vs. DSE in the study, with 254, 193 up-regulated genes and 187, 193 down-regulated genes in DSE compared to WJ and LP. GO analysis and KEGG signaling pathway analysis showed that DEGs are significantly related to contractile fiber, sarcolemma, and dystrophin-associated glycoprotein complex, myofibril, sarcolemma, and myosin II complex, Glycolysis/Gluconeogenesis, Propanoate metabolism, and Pyruvate metabolism, etc. In combination with functional annotation of DEGs, key genes such as *ENO3* and *JUN* were identified by PPI network analysis.

**Discussion:**

In conclusion, the present study revealed key genes including *DES, FLNC, PSMD1, PSMD6, PSME4, PSMB4, RPL11, RPL13A, ROS23, RPS29, MYH1, MYL9, MYL12B, TPM1, TPM4, ENO3, PGK1, PKM2, GPI*, and the unannotated new gene *ENSSSCG00000020769* and related signaling pathways that influence the difference in muscle growth and could provide a theoretical basis for improving pig muscle growth traits in the future.

## 1 Introduction

Pork accounts for 35% of global meat consumption. Demand for pork in China continues to increase as the country develops socially and economically. Therefore, it is imperative to improve the pork production and quality of domestic pig breeders. The genetic improvement of swine mainly focuses on the production and reproductive traits that have economic value, focusing in production traits (1). In order to meet the demand for pork, the growth rate is a crucial assessment indicator in pig breeding. Growth in pigs is a complex trait regulated by multiple genes and their regulatory networks. Studying the development and growth properties of skeletal muscle and combining it with modern molecular breeding techniques is an effective way to increase the efficiency of pig breeding.

Pig breeding resources are found all over the world (2). During the process of natural selection, Chinese indigenous pig breeds have exhibited numerous outstanding characteristics and significant phenotypic variations (3). Diannan small ears (DSE) pig is a small Chinese indigenous breed with slow growth and low adult weight (4). They are grown in the southern areas of Mengla, Ruili, and Yingjiang in Yunnan Province, which have a subtropical climate (5). DSE has numerous distinct phenotypic traits and are of superior commercial value (6, 7). DSE pork is abundant in nutrients and has a unique taste, and it has high stress resistance and adaptability to harsh environmental conditions (8). At the same time, DSE is also an ideal animal model for medical research. All these factors make DSE a valuable genetic resource. In addition, the research of genetic diversity and selection signatures within DSE shown it has lower genetic diversity than other breeds, indicating the importance of strengthening conservation strategies for DSE (9). At present, there are limited studies on the genetic improvement of muscle growth traits in DSE. It was therefore necessary to screen for key genes involved in pig muscle growth and development.

Wujin pig (WJ) is another excellent local breed found in the plateau regions of Yunnan, Sichuan, and Guizhou in China. It has valuable characteristics such as strong disease resistance, high fecundity, good mothering, strong adaptability, and high-quality pork products (10). As a typical fatty pig breed, WJ has become a famous high-quality ham product both domestically and internationally due to its delicious meat, abundant nutrition, elevated amino acid content, heavy collagen, and delicate flavor (11). WJ is broadly studied in China. It has strong fatty acid transport capacity and fat deposition rate in the body, and the expression of related genes involved in muscle cell differentiation and muscle growth and development is down-regulated compared with Landrace pig (LP) (12, 13). Western commercial pig breeds have consistently been selected for rapid muscle growth and development (14). LP is a typical lean pig breed originated in Denmark, with stable production performance and genetic stability, and is famous for its fast growth rate, strong feed utilization, and elevated lean meat rate (15). In the study conducted by Fang et al. (16), it was found that the rapid growth of LP may be attributed to the concentration of gene expression in muscle tissue on biological functions associated with muscle contraction.

Phenotypic differences are primarily determined by genetic differences (17). The growth rate and potential of skeletal muscle depends largely on the development of embryonic muscle fibers, with muscle fiber proliferation suspended between 85 and 95 days of gestation and not significantly altered by external conditions (18). Therefore, the disparity in the number of muscle fibers among various pig breeds may be permanent (19). This study used RNA-seq method to construct a transcriptome map of the Longissimus dorsi muscle (LDM) of pigs with different growth phenotypes. Comparison of differentially expressed genes (DEGs) in muscle growth between DSE, Chinese indigenous pig breed WJ, and introduced pig breed LP, and the screening of key candidate genes will provide a theoretical basis for future improvement of pig muscle growth traits.

## 2 Materials and methods

### 2.1 Animal and sample preparation

Animals under study were from two Chinese indigenous pig breeds (DSE pig, *n* = 3; WJ pig, *n* = 3) and one introduced pig breed (LP, *n* = 3). They were housed in similar environmental and nutritional conditions. All pigs were offered the same type of feed, and they were involved in the trial from around 30 kg of body weight (BW) until around 100 ± 2 kg BW. The animals were slaughtered according to the standard protocol of the Yunnan Agricultural University, and the LDM samples were collected after the slaughter. Samples were stored immediately in liquid nitrogen and later were stored at −80°C refrigerator for subsequent total RNA extraction. The research proposal and the relevant experimental procedures were approved by the institutional Animal Care and Use Committee of Yunnan Agricultural University (Case Number: 20210915).

### 2.2 Total RNA isolation from LDM samples

Total RNA was extracted from LDM tissues using the TRIzol Reagent kit (Invitrogen, San Diego, CA). The concentration and purity of the total RNA were checked using the NanoDrop 2000 Biophotometer and their completeness was verified by agarose gel electrophoresis.

### 2.3 Library preparation and RNA sequencing

A pool of equimolar ratios of RNAs from three individuals was established. The pooled RNA samples were purified with an RNeasy Micro Kit (Cat. Qiagen-74004; venlo, The Netherlands) for the preparation of complementary DNA (cDNA) library (approximately 3 μg of total RNA). Poly (A) messenger RNA (mRNA) isolation, first and second strand cDNA synthesis, fragment and adaptor ligation, and cDNA library preparation were performed sequentially with the TruSeq RNA Sample Prep Kit (Cat. RS-122-2002; Illumina, San Diego, CA). After that, each sample product was loaded onto the flow cell channels of the HighSeq 2000 platform (Illumina) for double-end 150 bp sequencing. The insert size of the double-ended library is 380 bp, and three biological replicas were included in each group.

### 2.4 Mapping and alignment of sequence reads

The raw reads obtained from RNA-seq were processed using CLC Genomics Workbench 4.8 (Qiagen), which trimmed the adapter sequences and removed low-quality reads. Pair-sequenced read files from each lane were separately mapped to the *Sus scrofa* reference genome (10.2) using Top Hat11, and two mismatches were allowed for 150-bp reads in each alignment.

### 2.5 Differential gene expression analysis using RNA-seq

The fragments per kilobase of transcript per million fragments mapped (FPKM) for all genes were calculated using Cuff software (version 2.1.2). Gene expression differences were evaluated using the Fisher exact test after normalizing the total number of mapped reads using the top-quartile normalization method. DEGs between the breeds were identified using the statistical significance criteria: | log2 fold change | > 1 and false discovery rate (FDR) < 0.01.

### 2.6 Functional annotation of genes with differential expression

Gene Ontology (GO) enrichment analysis of DEGs were performed using the GOseq software to determine their primary biological functions, and conducted Kyoto Encyclopedia of Genes and Genomes (KEGG) pathway enrichment analysis on DEGs. The GO terms and KEGG pathway enrichments with an adjusted *p*-value (Benjamini) less than 0.05 were considered significant.

## 3 Results

### 3.1 Summary of RNA-seq data

**Figure 1:**
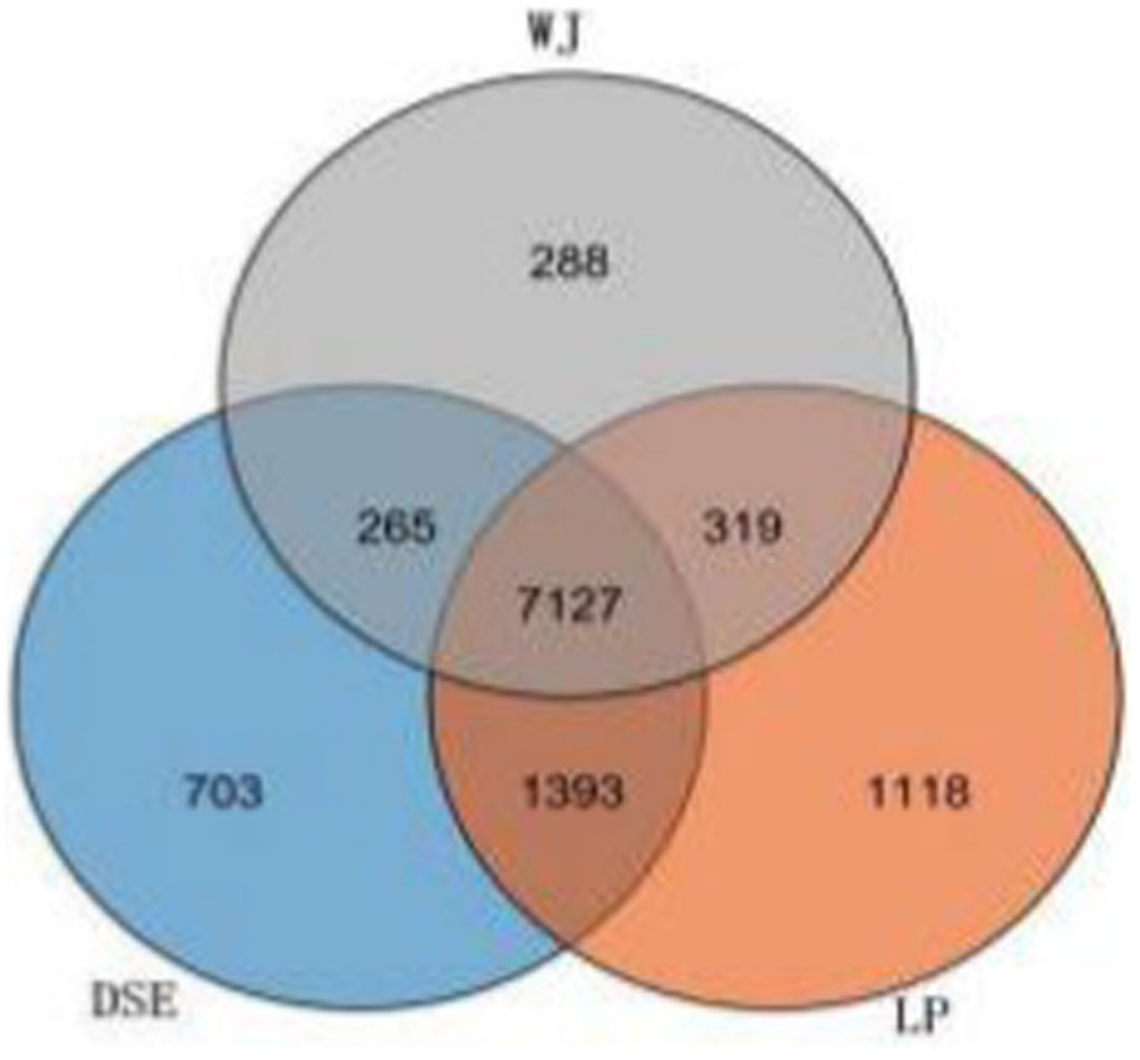
Venn diagrams of the number of genes expressed in each group. DSE, Diannan small ears pig; LP, Landrace pig; WJ, Wujin pig.

After removing the adaptor sequence and low-quality reads, each sample yielded clean reads ranging from 6,589,139 bp to 7,171,030 bp. Approximately 98% (ranging from 96% to 99%) of the clean reads were successfully mapped to the *Sus scrofa* reference genome (Supplementary Table S1). By calculating FPKM values as a function of gene expression levels, we observed that 11,213 genes are expressed in LDM tissue. Among these genes, 7,127 had the same expression across the three groups (Figure 1). The FPKM values of the expressed genes were mostly distributed between 0 ~ 0.1 and 0.3 ~ 15 (Supplementary Figure S1). The heat map of all co-expressed genes showed that the three biological repeats in each group were first clustered together, but the transcriptome patterns of DSE and WJ were different from those of LP (Supplementary Figure S2).

### 3.2 Analysis of differentially expressed genes

Using |log2 (fold change)| > 1 and FDR < 0.01 between groups as strict criteria, 441 DEGs were screened out when comparing DSE and WJ (Supplementary Table S2 and Supplementary Figure S3A), including 254 up-regulated and 187 down-regulated DEGs (Supplementary Table S2 and Supplementary Figure S3A). In the comparison of LP and DSE (Supplementary Table S3 and Supplementary Figure S3B), 339 DEGs were detected, including 146 up-regulated and 193 down-regulated DEGs; in the comparison of WJ and LP (Supplementary Table S4 and Supplementary Figure S3C), 695 DEGs were detected, including 296 up-regulated and 399 down-regulated DEGs. The number of DEGs in DSE vs. LP and DSE vs. WJ is higher than in WJ vs. LP, indicating that DSE is different from WJ and LP and has a unique pattern of gene expression.

**Figure 2:**
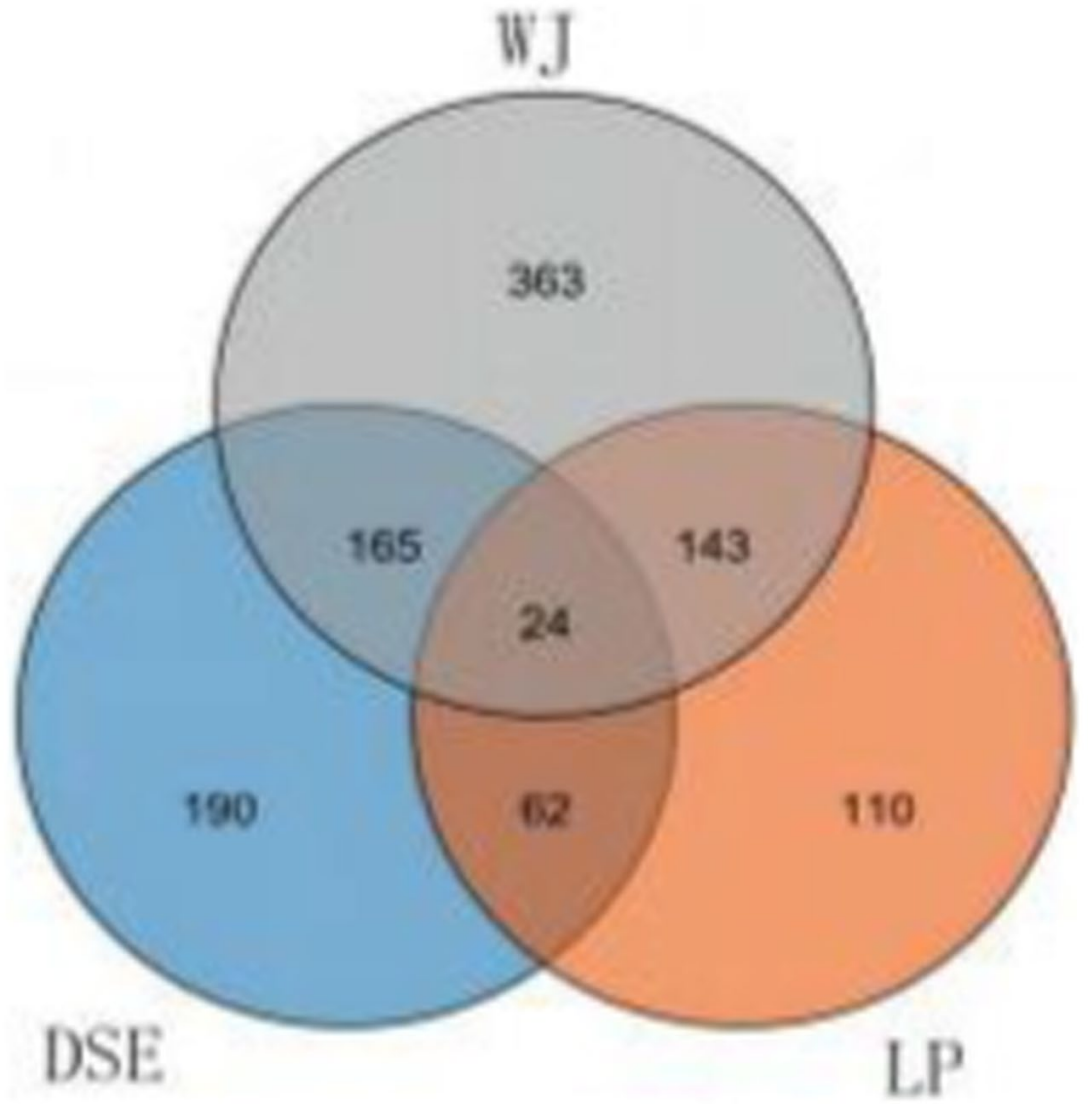
Venn diagrams of differentially expressed genes in the comparisons of LP vs. DSE, DSE vs. WJ, and WJ vs. LP. DSE, Diannan small ears pig; LP, Landrace pig; WJ, Wujin pig.

About 167 overlapping DEGs were found between WJ vs. LP and LP vs. DSE, indicating that LP has similar differences in prenatal muscles with WJ and DSE (Figure 2). A total of 189 DEGs were identified in the WJ pig LDM, while 86 DEGs were identified in DSE pigs. There are also 24 overlapping DEGs in three groups. In this experiment, 62 DEGs unique to the DSE were focused on. These DEGs are different from WJ and LP, which is helpful for additional studies of DSE.

### 3.3 Go functional annotation of the differentially expressed genes

**Figure 3:**
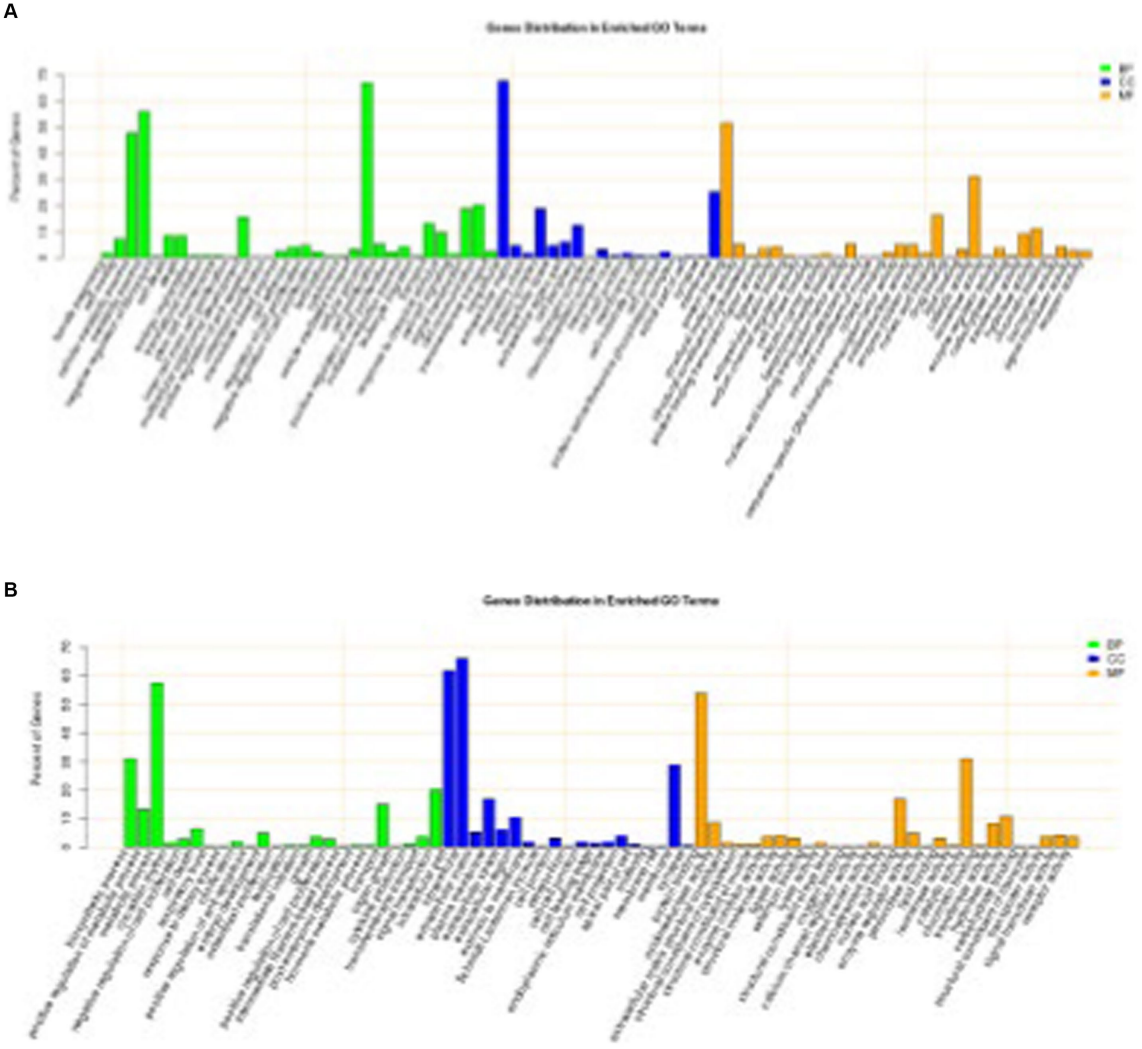
Significantly enriched GO for differentially expressed genes. **(A)** DSE vs. WJ, **(B)** LP vs. DSE. DSE, Diannan small ears pig; LP, Landrace pig; WJ, Wujin pig.

Of the 441 DEGs between DSE and WJ, 361 were annotated. A total of 330 terms (Supplementary Table S5) were significantly enriched in the three categories of cellular components, biological processes, and molecular functions (*p* < 0.05). Among them, the top 20 terms (the 20 terms with the lowest *p* value) consist of 12 cellular components, 4 biological process terms, and 4 molecular function terms (Figure 3A). There are 15 terms (Table 1) related to muscle growth and development, including contractile fiber (GO:0043292), sarcolemma (GO:0042383), and dystrophin-associated glycoprotein complex (GO:0016010), etc.

**Table 1:** The significantly enriched associated with muscle growth and development.

Groups	Term ID	Description	*P* value	Gene number
DSE vs. WJ	GO:0043292	Contractile fiber	0.001404052	8
	GO:0042383	Sarcolemma	0.001975232	5
	GO:0016010	Dystrophin-associated glycoprotein complex	0.002108138	3
	GO:0051918	Negative regulation of fibrinolysis	0.003448377	2
	GO:0010761	Fibroblast migration	0.003518878	3
	GO:0030016	Myofibril	0.003640074	7
	GO:0007517	Muscle organ development	0.013446942	10
	GO:0048644	Muscle organ morphogenesis	0.017825975	4
	GO:2000098	Negative regulation of smooth muscle cell-matrix adhesion	0.022762245	1
	GO:0035994	Response to muscle stretch	0.024443189	1
	GO:0003779	Actin binding	0.032651412	9
	GO:0008092	Cytoskeletal protein binding	0.040131442	15
	GO:0003221	Right ventricular cardiac muscle tissue morphogenesis	0.046140655	1
	GO:0048741	Skeletal muscle fiber development	0.054626439	4
	GO:0060415	Muscle tissue morphogenesis	0.059398223	3
LP vs. DSE	GO:0030016	Myofibril	2.70E-10	14
	GO:0042383	Sarcolemma	4.39E-06	7
	GO:0016460	Myosin II complex	0.00031004	3
	GO:0008092	Cytoskeletal protein binding	0.001456212	16
	GO:0060537	Muscle tissue development	0.001561886	11
	GO:0008307	Structural constituent of muscle	0.001578736	3
	GO:0005515	Stress fiber	0.002736095	4
	GO:0048738	Cardiac muscle tissue development	0.005361607	6
	GO:0003779	Actin binding	0.006301305	9
	GO:0007517	Muscle organ development	0.006948182	9
	GO:0055020	Positive regulation of cardiac muscle fiber development	0.014456304	1
	GO:0016010	Dystrophin-associated glycoprotein complex	0.01729993	2
	GO:0007519	Skeletal muscle tissue development	0.017980988	6
	GO:0035994	Response to muscle stretch	0.018030091	1
	GO:0014809	Regulation of skeletal muscle contraction by regulation of release of sequestered calcium ion	0.018215829	1
	GO:0050115	Myosin-light-chain-phosphatase activity	0.018267419	1	
	GO:0042692	Muscle cell differentiation	0.018328758	8
	GO:0048661	Positive regulation of smooth muscle cell proliferation	0.024277525	2
	GO:0015629	Actin cytoskeleton	0.024573178	9
	GO:0060415	Muscle tissue morphogenesis	0.031254293	3
	GO:0006941	Striated muscle contraction	0.03572248	3
	GO:0055007	Cardiac muscle cell differentiation	0.040803718	3
	GO:0005859	Muscle myosin complex	0.053823853	1

DSE, Diannan small ears pig; LP, Landrace pig; WJ, Wujin pig.

Of the 339 DEGs between LP and DSE, 278 were annotated. A total of 496 terms (Supplementary Table S6) were significantly enriched in the three categories of cellular components, biological processes, and molecular functions (*p* < 0.05). Among them, the top 20 terms consist of 9 cellular components, 9 biological processes, and 2 molecular functions (Figure 3B). There are 23 terms (Table 1) related to muscle growth and development, including myofibril (GO:0030016), sarcolemma (GO:0042383), and myosin II complex (GO:0016460), etc.

### 3.4 KEGG functional annotation of the differentially expressed genes

**Figure 4:**
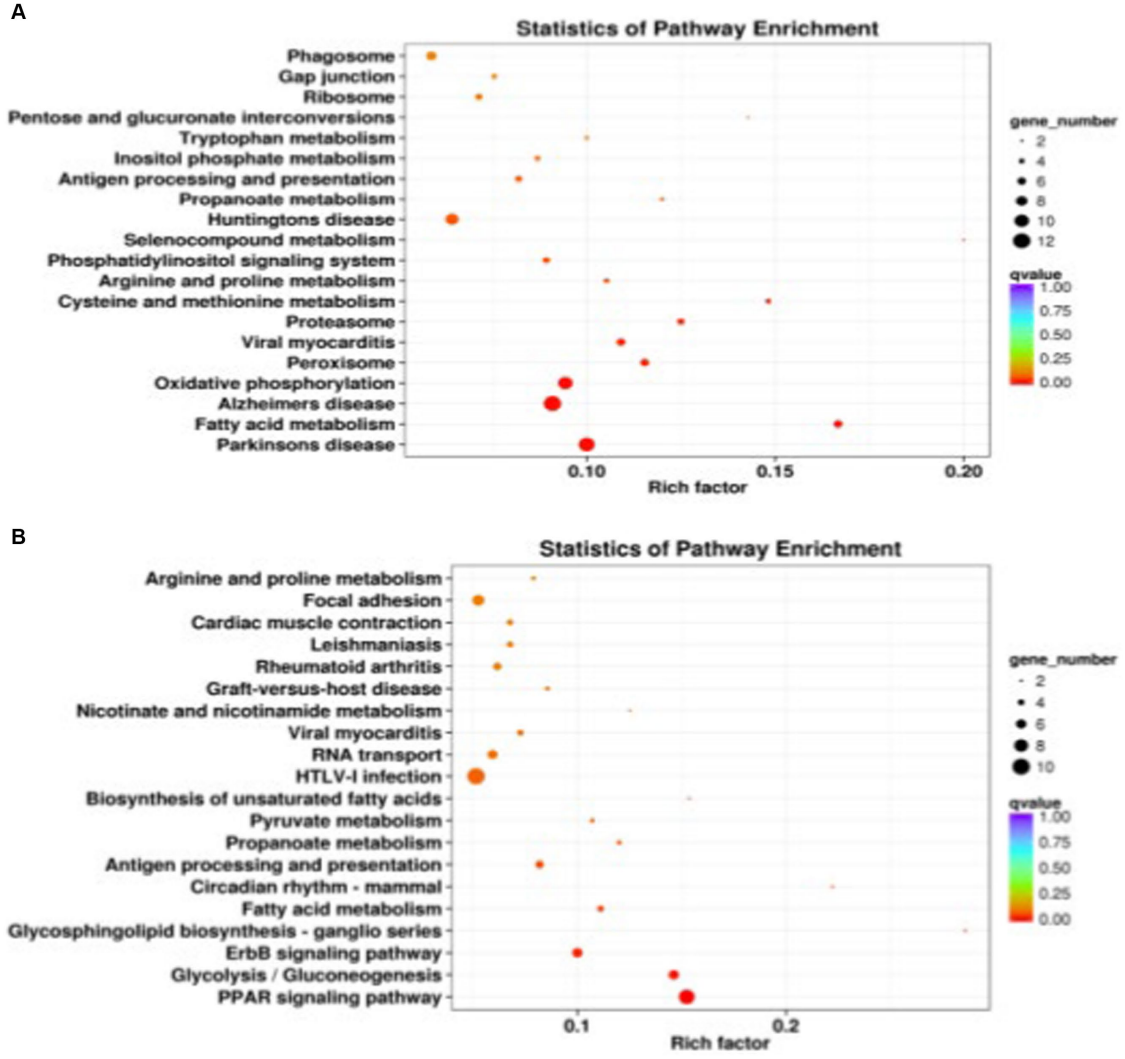
Significantly enriched KEGG for differentially expressed genes. **(A)** DSE vs. WJ, **(B)** LP vs. DSE. DSE, Diannan small ears pig; LP, Landrace pig; WJ, Wujin pig.

A total of 441 DEGs between DSE and WJ were integrated into the KEGG pathway database. Among these, 13 pathways (*p* < 0.05) were found to be significantly enriched (Supplementary Table S7 and Figure 4A). Muscle growth and development involve three pathways (Table 2), including the Proteasome pathway, Cysteine and Methionine metabolism, and Propanoate metabolism. 339 DEGs between LP and DSE were integrated into the KEGG pathway database. A total of 10 pathways (*p* < 0.05) were significantly enriched (Supplementary Table S8 and Figure 4B). Muscle growth and development involve three pathways (Table 2), including Glycolysis/Gluconeogenesis, Propanoate metabolism, and Pyruvate metabolism.

**Table 2:** The significantly enriched pathways associated with muscle growth and development.

Groups	Pathway ID	Description	*P* value	Gene number
DSE vs. WJ	ssc03050	Proteasome	0.0102129521668	5
	ssc00270	Cysteine and methionine metabolism	0.011720118035	4
	ssc00640	Propanoate metabolism	0.0494126559507	3
LP vs. DSE	ssc00010	Glycolysis / Gluconeogenesis	0.00116100335695	6
	ssc00640	Propanoate metabolism	0.0358095343245	3
	ssc00620	Pyruvate metabolism	0.0478451377886	3

DSE, Diannan small ears pig; LP, Landrace pig; WJ, Wujin pig.

There are 20 genes significantly enriched in Table 3 that are involved in muscle growth and development. Among them, six genes (DES, FLNC, RPL11, MYH1, MYL9, and TPM1) were highly enriched in GO terms and significantly enriched in the KEGG pathway. In addition, *PSMD1, PAMD6, PSME4, PSMB4, ENSSSCG000020769, RPL13A, RPS23, RPS29, TPM4, ENO3, PGK1, GPI,* and *PKM2* were found to be enriched only in the KEGG pathway, while MYL12B was enriched only in the GO term.

**Table 3:** Information of 20 DEGs associated with muscle growth and development.

Groups	Gene name	log2.Fold_change.	P value	Descriptions
DSE vsWJ	DES	1.32019928743822	0	Protein binding, contractile fiber, sarcolemma, cytoskeletal protein binding
	FLNC	−1.34055685591147	3.77813642228202E-161	Protein binding, sarcolemma, actin binding, cytoskeletal protein binding
	PSMD1	1.31482666409046	1.78E-16	Proteasome
	PSMD6	1.51884055611205	0.000129176587534192	Proteasome
	PSME4	1.06776523373258	1.98235400244217E-21	Proteasome
	PSMB4	2.03341372894181	6.75E-06	Proteasome
	ENSSSCG00000020769	0.984504128460864	2.87E-06	Proteasome
	RPL11	0.995278600055038	3.56720719523283E-06	Ribosome, structural constituent of ribosome
	RPL13A	−4.77530352432005	0.000210928298417015	Ribosome
	RPS23	1.0334137289418	1.82700093638566E-12	Structural constituent of ribosome, Ribosome
	RPS29	0.914577499431955	7.01387929945769E-09	Structural constituent of ribosome, Ribosome
LP vs. DSE	MYH1	1.75837231597449	0	Muscle myosin complex
	MYL9	1.47785378627881	0.0000190204641544921	Myosin II complex, stress fiber, Regulation of actin cytoskeleton
	MYL12B/MRLC2	−2.22561189263407	0	Myosin II complex, stress fiber
	TPM1	1.55308343700939	3.77813642228202E-161	Myofibril
	TPM4	1.22476456771945	1.78E-16	Cardiac muscle contraction
	ENO3	1.44743845208445	0.000129176587534192	Glycolysis / Gluconeogenesis
	PGK1	1.36777321091522	1.98235400244217E-21	Glycolysis / Gluconeogenesis
	PKM2	1.86206226303013	6.75E-06	Protein binding, Glycolysis / Gluconeogenesis, Pyruvate metabolism
	GPI	1.95312868602623	2.87E-06	Glycolysis / Gluconeogenesis

DSE, Diannan small ears pig; LP, Landrace pig; WJ, Wujin pig.

### 3.5 PPI network construction and hub gene identification

**Figure 5:**
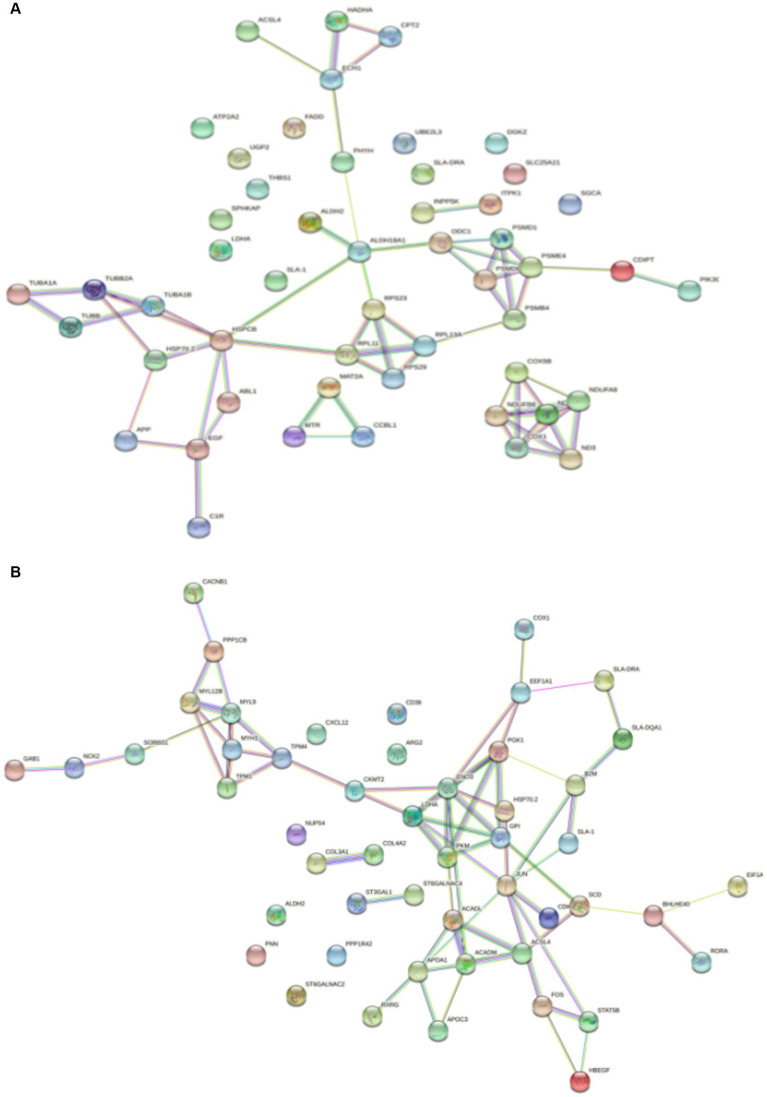
Significantly enriched PPI for differentially expressed genes. **(A)** DSE vs. WJ, **(B)** LP vs. DSE. DSE, Diannan small ears pig; LP, Landrace pig; WJ, Wujin pig.

Protein–Protein Interaction (PPI) network analysis was performed on the key genes among the screened DEGs to gain a better understanding of their interactions. In the PPI network of DSE vs. WJ, a total of 51 nodes and 61 edges were established (Figure 5A). Additionally, in the PPI network of LP vs. DSE, a total of 50 nodes and 65 edges were established (Figure 5B). In Figure 5A, we found four distinct clusters in the network, consisting of proteins encoded by DEGs. In Figure 5B, most of the proteins in the network interacted with multiple different partners. However, we found that the proteins enolase 3 (ENO3) and Jun proto-oncogene, AP-1 transcription factor subunit (JUN) have the highest degree of connectivity (eight edges), which may enable them to play a role as “hub” proteins, acting as controllers within biochemical pathways.

## 4 Discussion

RNA-seq provides a quantitative and open system for large-scale analysis of transcription results (20). In the animal husbandry industry, meat quality are critical economic characteristics (21). Identifying key candidate genes involved in muscle growth is effective in studying molecular genetic regulatory mechanisms for muscle growth and development. This promotes muscle growth and provides people with a high-quality source of meat. Muscle is an essential part of all meat products. Growth performance and product quality are considered to be the basic conditions for modern breeding (22). Muscle growth is regulated by the homeostatic balance of muscle protein biosynthesis and degradation (23, 24). This is the result of a combination of a distinctive genotype, nutrient status, age, and muscle type in muscle tissue (25). In the study, 11,213 genes were detected in the longest muscles. Moreover, by analyzing the GO and KEGG signaling pathways of selected DEGs, significant functional genes and signaling pathways associated with phenotypic differences between DSE, WJ, and LP were found. A large proportion of previously screened DEGs are involved in metabolic regulation and muscle development. A total of 199 DEGs were classified as associated with muscle growth and development at the time of GO term analysis, representing approximately 25.5% of the total number of DEGs. Among them, 20 DEGs have been identified as key regulators of myocyte differentiation and muscle development. These genes are thought to affect postnatal muscle growth rates, muscle fiber diameter and density, and fiber type.

Intermediate filaments are the main components of the cytoskeleton, and desmin and filament proteins play important roles in skeletal muscle (26). The Desmin (*DES*) gene encodes desmin, which is a tissue-specific type III intermediate filament expressed in skeletal muscle cells, cardiomyocytes, and smooth muscle cells (27). *DES* has been found to be related to striated muscle disease (28). *DES* connects myofibrillar structures such as sarcomeres, nuclei, mitochondria, and lysosomes around the Z-disc. This connection is essential for maintaining the lateral organization and alignment of sarcomere structures during myofibril regeneration (29). Studies have shown that *DES* can effectively serve as a marker for muscle fiber regeneration (30). Filamins (FLNs) are large dimeric actin-binding proteins that regulate actin cytoskeleton remodeling (31). FLNs are primarily expressed in skeletal muscle and cardiac muscle, and they are located in various structures such as the Z-disc, myotendinous junctions, myotomes, and intercalated discs (32). They are involved in the organization and stabilization of the actin cytoskeleton, as well as in anchoring adhesion sites to transmembrane proteins, which play a role in cell-matrix and cell–cell interactions (33). Fujita et al. (34) found that Filamin C (FLNC) plays an indispensable role in maintaining the structural integrity of cardiac and skeletal muscles, which supports their resistance to stress. In addition, studies have also found that mutations in the FLNC gene can cause hypertrophic cardiomyopathy (35). The results of this study indicate that the expression levels of the DES gene are significantly higher in DSE than in WJ, while the expression levels of the *FLNC* gene are lower in DSE than in WJ. This result may be due to the significant difference in adult body weight between the two local pig breeds.

The formation of muscle fibers, or myogenesis, is believed to be a complex process involving the proliferation of myoblasts and the increase of structural proteins. This process is initiated by mesenchymal stem cells that originate from mesoblasts (36, 37). Myosin is the structural component of muscle (38). The myoglobin II molecule is an essential component of both muscle cells and non-muscle cells. They consist of two Myosin heavy chain (MyHC) and two Myosin light chain (MyLC) molecules (39, 40). The formation of muscle fibers is precisely regulated at the molecular level by the genes of muscle cells. Musclin heavy chain 1 (MyH1), also known as MyHC II.X, a type of muscle fiber found in fast-twitch muscles. Type II.X belongs to the transition type of oxidation in yeast-type, while the proportion of oxidized muscle fibers is positively related to meat quality (41). In addition, the *MyH1* gene is significantly associated with muscle contraction and muscle development (42). Muscle growth inhibition in muscle differentiation can help slow down and inhibit the expression of rapidly condensed heavy chain genes, as well as have some effects on DNA repair (43). Although MyLC is not in the myosin protein family, it plays an important role in myogenase catalysis supramolecular complex formation (44). Among them, MyL9 is a key link for activating muscle global activity to provide power for cells (45). MYL12B regulates the activity of myosin light chain ATP enzyme through phosphorylation (46). Tropomyosin is an essential regulatory protein during muscle contraction. It mainly regulates the contraction of horizontal and smooth muscles (47). *TPM1* and *TPM4* genes are members of this family (48). In the study, mRNA expression levels of the genes *MYH1, MYL9, TPM1*, and *TPM4* were lower in DSE than in LP. In turn, the expression of the *MYL12B* gene is higher in DSE than in LP. Protein is a manifestation of life functions (49). The proteasome is responsible for the degradation of most proteins in the cell through the ubiquitin-proteasome system (UPS) of the protein-protease system (50). The protease complex is composed of one 20S core particles (CP) and two 19S regulatory particles (RP). In the study, the mRNA expression levels of the genes *PSMD1, PSMD6, PSME4,* and *PSMB4* were higher in DSE than in WJ. A new gene, *ENSSSCG000020769*, was found to be involved in proteasome synthesis. Its expression level is higher in DSE than in WJ.

It is well accepted that total protein synthesis in muscle is related to both the ribosome content of the tissue and the activity per ribosome (51). The increase in protein synthesis may not be solely attributable to an increase in ribosomal and mRNA production in muscle. However, the high ribosome concentration may be associated with the rapid rate of protein synthesis (52, 53). The ribosome is a venue for protein synthesis and is involved in cell proliferation, withering and differentiation. Ribosomal protein (RP) is the main component, named ribosomal protein large (RPL) and ribosomal protein Small (RPS) according to the source size sub-tadlies (54). In the study, mRNA expression levels of the genes *RPL11, RPS23,* and *RPS29* were higher in DSE than in WJ. The results show that protein synthesis and metabolism related genes in WJ are down-regulated compared to LP, in particular the ribosomal pathway and several ribosomal protein genes.

Muscle fiber is considered an essential factor influencing numerous biochemical processes and thereby meat quality (55). Intensive selection for lean growth in modern pig breeds has resulted in a shift in muscle metabolism towards more glycolytic and less oxidative fiber types. A high proportion of type II fiber adversely affects meat quality, while oxidative-type fiber generally contains higher intramuscular fat (55, 56). Enolase (ENO) is a kind of glycoling enzyme, participating in the regulation of the energy metabolic pathway of glycolysis and gluconeogenesis (57). Energy metabolism is thought to play an important role in skeletal muscle growth and development, directly affecting muscle fiber type and meat quality. ENO3 is positioned in muscle tissue, expressed in proliferative muscle cells and differentiated muscle pipes (58). Lactate dehydrogenase (LDH) is the hallmark enzyme of glycolysis (59). LDHA is abundantly expressed in skeletal muscle and is mainly responsible for catalyzing lactate and oxidizing NADH to generate NAD+ (60). The reaction catalyzed by phosphoglycerate kinase (PGK) is the first energy-producing reaction in glucose breakdown and is an important metabolic enzyme in the glycolysis pathway (61). Mutations in the *PGK1* gene can lead to reduced glycerol kinase activity and interfere with normal glucose disassembly pathways and energy production (62). Glucose can be utilized intracellularly through conversion to hexokinase 2 (HK-2), phosphofructokinase (PFKM), and pyruvate kinase M (PKM). Pyruvate kinase muscle isoenzyme 2 (PKM2) is an isoenzyme of the glycolytic enzyme pyruvate kinase (63). PKM2 and its alternatively spliced mRNA (PKM1) are both produced by the *PKM* gene (64). *PKM1* is mainly expressed in tissues with elevated energy demands, such as muscle and brain (65), while *PKM2* is expressed at varying levels in most cell types (66). But *PKM2* reduces pyruvate kinase activity and promotes the pentose phosphate pathway (PPP), thus preventing oxidative stress (67). Glucose-6-phosphate isomerase (GPI) is a gene-encoded product that plays an important regulatory role in cell growth, differentiation, and apoptosis (68). In this study, the expression levels of ENO3, PGK1, PKM2 and GPI genes were lower in DSE than in LP.

The WJ has a high body fat content, especially in terms of intramuscular fat, which is higher than other pig breeds (11). DSE is known for its superior meat quality and higher fat deposits compared to Western pig breeds (8). In a previous report by this experimental team, it was shown that the increasing mechanism of intramuscular fat (IMF) content deposition in fatty pigs may be due to higher lipogenesis and fatty acid transport capacity and lower lipolysis capacity (13). Stearoyl-CoA desaturase (SCD) is a regulatory enzyme produced by fat (69) that produces monounsaturated fatty acids (MUFA) that contribute to cell growth, survival, differentiation, metabolic regulation, and signaling (70). Long-chain ACYL-CoA synthase 4 (ACSL4) is an important catalytic enzyme for the preparation of polyunsaturated fatty acids (PUFA). It is present in anabolic steroid tissues, plays an important role in the anabolism and catabolism of fatty acids, and is important for growth and developmental processes, also showing a regulatory role (71, 72). Acyl-CoA dehydrogenase (ACAD) is a mitochia lutease family involved in the metabolism of fatty acids and branches (73). Acyl-CoA dehydrogenase medium chain gene (*ACADM*) and Acyl-COA dehydrogenase long chain gene (*ACADL*) can catalyze the mitochondrial oxidation of straight-chain fatty acid mitochondria. In this study, the top 20 GO terms included terms related to triglyceride metabolism processes, cholesterol metabolism processes, negative regulators of fatty acid biosynthesis, and fatty acid biosynthesis processes. In the KEGG functional annotation we found pathways involved in IMF synthesis in the body, like the PPAR signaling pathway, fatty acid metabolism pathway, propionate metabolism pathway, pyruvate metabolism pathway, and biosynthetic unsaturated fatty acid pathway. Based on the PPI analysis, the ACASL4, ACADM and ACADL genes were identified as the three key nodes with higher expression levels in DSE compared to LP. Considering the strong fat deposition ability of Chinese native pigs, this result could explain the slower growth rate of DSE compared to other exotic pig breeds.

## 5 Conclusion

In summary, this study identified 11,213 genes in the LDM tissue in pigs. By comparing three different pig breeds (WJ, LP, and DSE), we found 62 unique DEGs. Among them, the genes *DES, FLNC, PSMD1, PSMD6, PSME4, PSMB4, RPL11, RPL13A, ROS23, RPS29, MYH1, MYL9, MYL12B, TPM1, ENO3, PGK1, PKM2, GPI,* and the unannotated new gene *ENSSSCG201020769* were identified as key genes that regulate muscle growth and development. These genes determine the pig’s postnatal growth rate, muscle fiber diameter and density, and fiber type. The research results shown here, provide a theoretical basis for improving pig muscle growth traits in the future.

## Data availability statement

The data presented in the study are deposited in the NCBI repository, accession numbers PRJNA1050857 and SAMN38755984-SAMN38755986.

## Ethics statement

The animal study was approved by Animal Care and Use Committee of Yunnan Agricultural University (Case Number: 20230906). The study was conducted in accordance with the local legislation and institutional requirements.

## Author contributions

QL: Conceptualization, Data curation, Formal analysis, Investigation, Methodology, Validation, Visualization, Writing – original draft, Writing – review & editing. MH: Conceptualization, Investigation, Validation, Writing – original draft, Writing – review & editing. JZ: Investigation, Software, Writing – review & editing. LY: Investigation, Software, Writing – review & editing. WC: Formal analysis, Investigation, Writing – review & editing. YX: Investigation, Writing – review & editing. ZS: Data curation, Funding acquisition, Methodology, Resources, Supervision, Writing – review & editing.
