# Supplementary figures and images for: Comparison of differentially expressed genes in longissimus dorsi muscle of Diannan small ears, Wujin and landrace pigs using RNA-seq
Source: Front Vet Sci. 2024 Jan 5;10:1296208. doi: 10.3389/fvets.2023.1296208 (PMC10796741; doi:10.3389/fvets.2023.1296208)

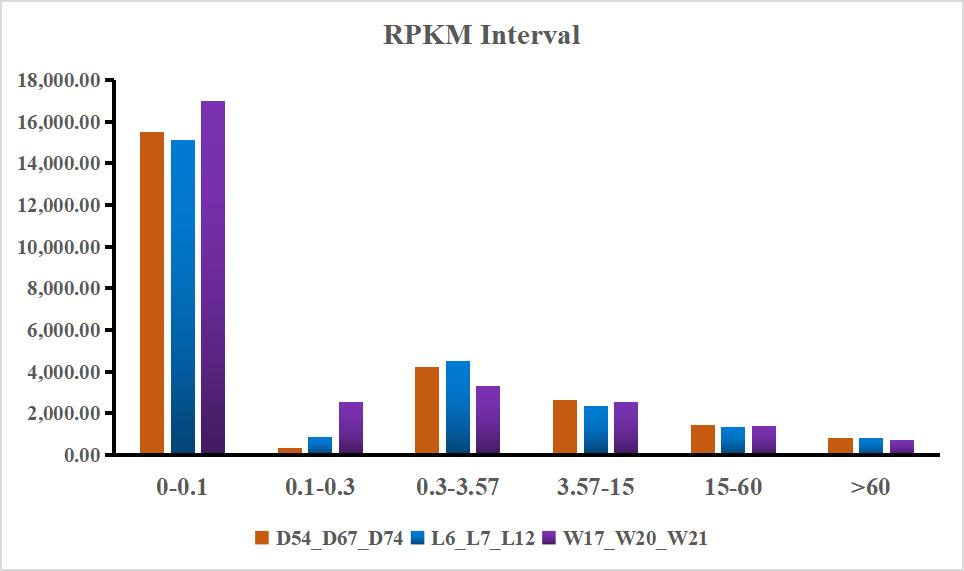

Supplement: Supplementary file 1 [file Figure_1.JPEG]

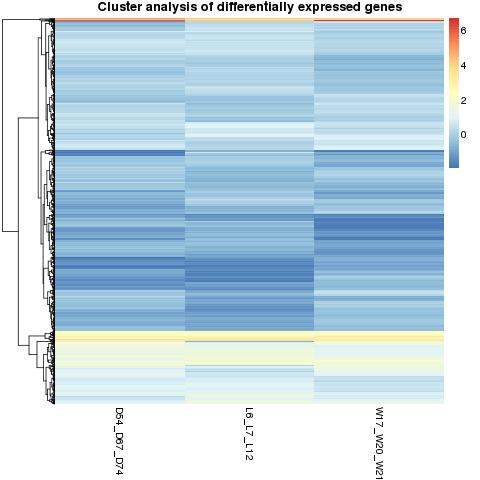

Supplement: Supplementary file 2 [file Figure_2.JPEG]

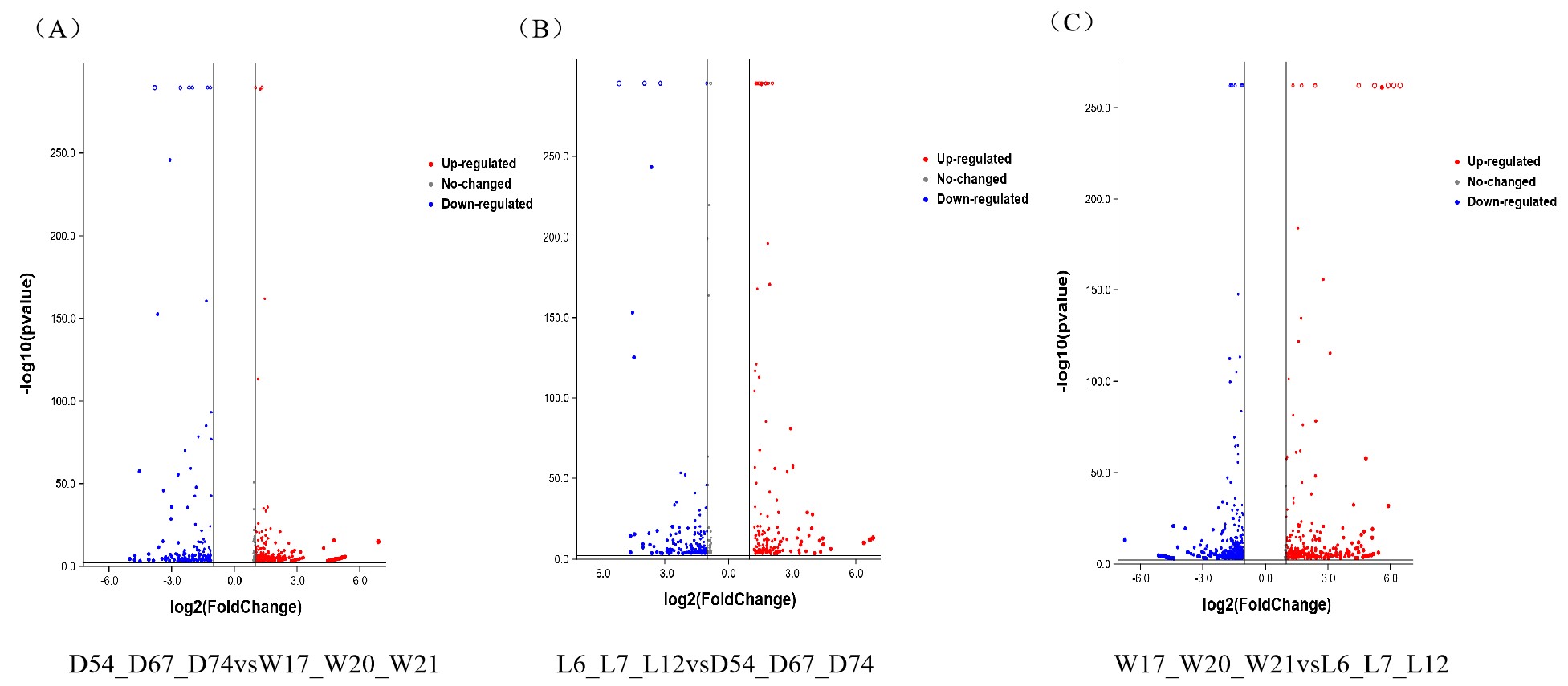

Supplement: Supplementary file 3 [file Figure_3.JPEG]
